# Biofilm mechanics: Implications in infection and survival

**DOI:** 10.1016/j.bioflm.2019.100017

**Published:** 2019-12-19

**Authors:** Erin S. Gloag, Stefania Fabbri, Daniel J. Wozniak, Paul Stoodley

**Affiliations:** a Department of Microbial Infection and Immunity, The Ohio State University, Columbus, OH, 43210, USA; b Perfectus Biomed, SciTech Daresbury, Cheshire, WA4 4AB, UK; c Department of Microbiology, The Ohio State University, Columbus, OH, 43210, USA; d Department of Orthopedics, The Ohio State University, Columbus, OH, 43210, USA; e National Biofilm Innovation Centre (NBIC) and National Centre for Advanced Tribology at Southampton (nCATS), University of Southampton, Southampton, SO17 1BJ, UK

**Keywords:** Biofilm, Mechanics, Viscoelasticity, Biophysics, Extracellular matrix, Extracellular polymeric substance, Tolerance, Persistence

## Abstract

It has long been recognized that biofilms are viscoelastic materials, however the importance of this attribute to the survival and persistence of these microbial communities is yet to be fully realized. Here we review work, which focuses on understanding biofilm mechanics and put this knowledge in the context of biofilm survival, particularly for biofilm-associated infections. We note that biofilm viscoelasticity may be an evolved property of these communities, and that the production of multiple extracellular polymeric slime components may be a way to ensure the development of biofilms with complex viscoelastic properties. We discuss viscoelasticity facilitating biofilm survival in the context of promoting the formation of larger and stronger biofilms when exposed to shear forces, promoting fluid-like behavior of the biofilm and subsequent biofilm expansion by viscous flow, and enabling resistance to both mechanical and chemical methods of clearance. We conclude that biofilm viscoelasticity contributes to the virulence of chronic biofilm infections.

## Introduction

**Fig. 1:**
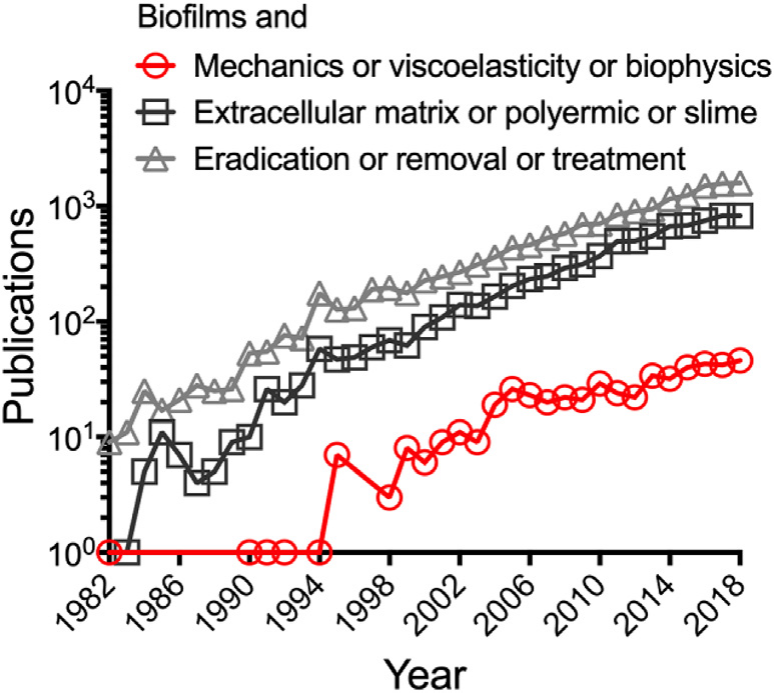
**Comparison of publication numbers between 1982**–**2018.** Publication numbers. Keywords indicated in the legend were searched using the Scopus database. Publication numbers for each keyword search from 1982, being the earliest identified biofilm mechanical paper, to December 2018.

Biofilms are usually considered from the physiological perspective of the encased microbial cells. However, they can also be considered as biophysical materials, where the cells are equivalent to colloids and the encasing extracellular polymeric slime (EPS) as a cross-linked polymer gel. This framework has allowed parallels to be drawn from soft matter physics, permitting the current understanding of biofilms as viscoelastic materials [[1], [2], [3]]. Despite recent interest in biofilm mechanics, it remains an under-represented research area compared to other focuses, such as biofilm eradication and EPS production (Fig. 1). This is largely twofold; firstly, the importance of mechanics to the survival and control of biofilms has only recently been recognized, and as such many microbiology research laboratories lack the equipment and expertise for these analyses. Secondly, biofilms are difficult to analyze mechanically, as they are microscopic and highly variable, both within and between biological replicates and species. Furthermore, biofilms are grown either as aggregates or attached at surface interfaces, and transferring biofilms to conventional mechanical testing equipment for this analysis, without disrupting them, can be challenging [4]. This is compared to materials routinely analyzed mechanically, which tend to be abiotic, uniform, not restricted in volume and relatively easy to handle. However, with technical advances, and the ingenuity of researchers to adapt novel methods, this hurdle is slowing being overcome, accounting for the steady rise of this field since the mid 1990’s (Fig. 1).

Understanding biofilm mechanical properties, and how the biofilm responds to mechanical forces in its surroundings, offers new insight into the establishment and survival of these microbial communities. Measuring a biofilm’s mechanical moduli (i.e. Young’s, shear, storage or loss; discussed below) offers a way of describing biofilms that is complementary to current quantification parameters from microscopy analysis, such a biomass or roughness. Quantifying parameters from microscopy analysis is subject to variation due to microscope settings and threshold levels. Biomass quantified in this manner is also likely to be an underestimate as not all biofilm EPS components can be stained, and subsequently visualized.

The external forces that biofilms experience during development, shape the structure and collective behavior of these communities. Therefore, biofilm mechanics is an important research focus from both a basic biology standpoint, but also has important consequences to infection and industryText box 1.

## Mechanical measurements of biofilms

**Fig. 2:**
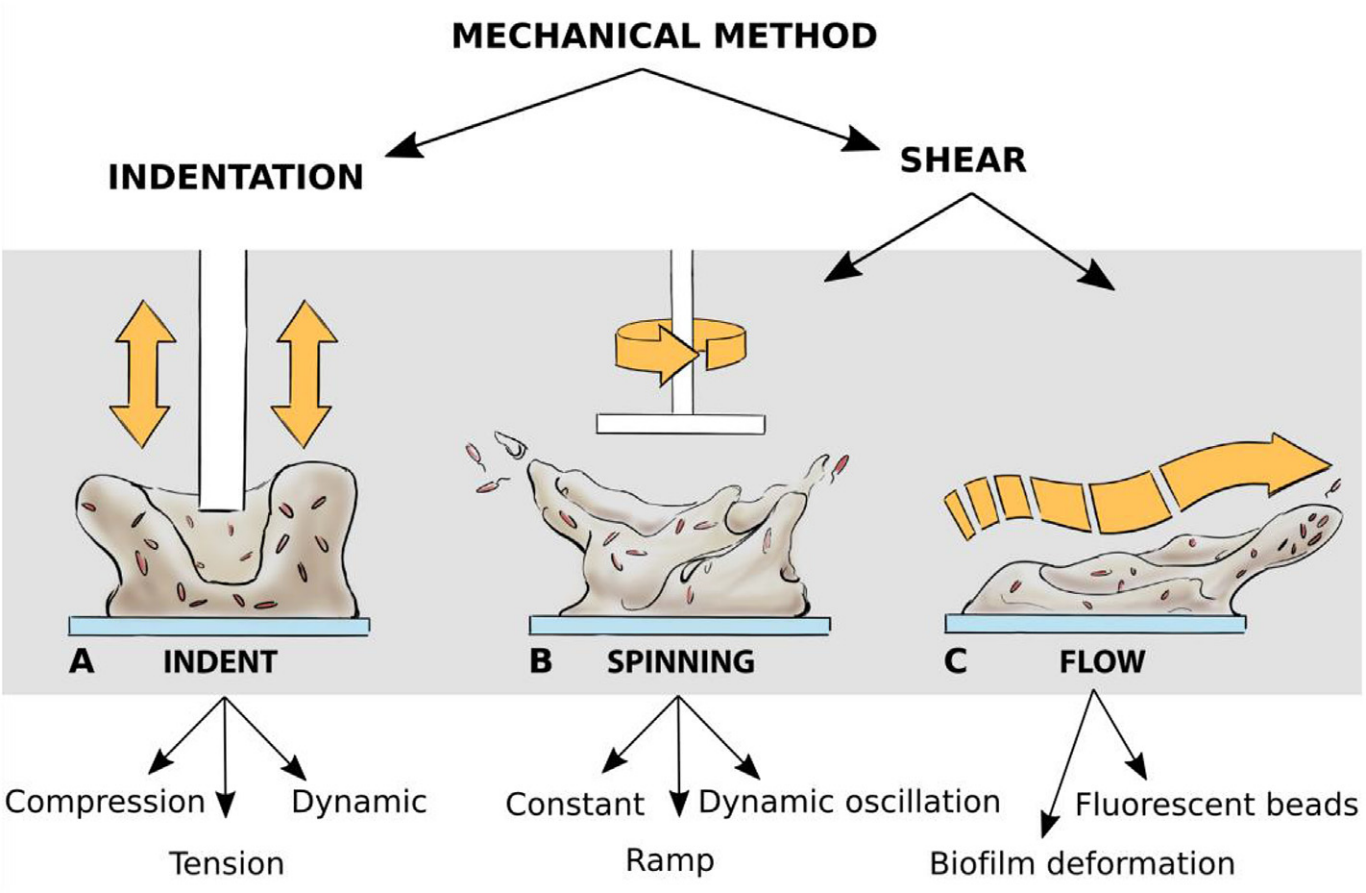
**Schematic of the different mechanical methods that can be used to analyze biofilms.** Mechanical methods to analyze biofilms can be divided into indentation (application of a normal force) or shear (application of a shear force). **(A)** For indentation, a probe or indenter is brought into contact with the biofilm, and the mechanical properties analyzed using either compression (pushing), tension (pulling) or dynamic (cycling of compression and tension) modes. For analysis under shear, there are two main ways that shear forces can be applied to a biofilm, **(B)** using spinning disc rheometry (spinning) or **(C)** using fluid shear (flow). **(B)** For spinning disc rheometry, a parallel plate probe is brought into contact with the biofilm, and changes in stress or strain measured under different modes of analysis. This can be either constant, where a constant stress (creep) or constant strain (relaxation) is applied; ramp, where an increasing stress or strain (stress-strain curve) is applied; or dynamic oscillation, where the probe is oscillated at either increasing stress, strain or frequency (frequency sweep, stress sweep, strain sweep). **(C)** When grown under fluid shear, time-lapse microscopy can be used to visualize and measure the deflection of biofilm structures in response to changes in the fluid flow rate. Alternatively, fluorescent beads can be incorporated into the system and their movement tracked through the biofilm.

There are several ways that the mechanical properties of biofilms can be analyzed, the most common are mechanical indentation or the application of shear stresses. Using these two methods, a number of different mechanical analyses can be performed (Fig. 2; Text box 1). Those pertinent to this review will be briefly discussed here, however for a more compressive review of mechanical methodology refer to Refs. [[5], [6], [7], [8]]. For analyses using mechanical indentation, a *normal force* is applied to the biofilm surface and can be analyzed both at the macro (rheometer or indenter) and micro (atomic force microscopy) scale (Fig. 2A). For analyses using shear stresses, a *shear force* is applied to the biofilm surface using either spinning disk rheology or flow cell systems (Fig. 2B and C). The latter can also be analyzed at the macro (measuring changes in biofilm deformation with changes in fluid shear rate) and micro (tracking the movement of fluorescently labeled beads through the biofilm) scale (Fig. 2C). From these different mechanical analyses various *moduli* can be calculated, which describe intrinsic mechanical properties of a given material [9,10].

Text box 1 *Normal or axial force:* A force that is applied perpendicular to a surface.

*Compression:* A force which pushes against a material to be tested.

*Tension:* A force which pulls a material apart. For this test the material needs to be adhered to the pulling surface.

*Shear force*: A force that is applied parallel to a surface.

*Stress*: The force applied over a unit area; (force/area).

*Strain*: Shape change or physical deformation of a material relative to the original dimensions of the material; dimensionless.

*Modulus*: The relationship between stress and strain that materials experience during mechanical measurements. This relationship can be linear, nonlinear or history dependent; (stress/strain).

*Oscillatory or dynamic test:* A test in which a force or strain applied to a material, in normal or shear, is done so in an oscillatory manner (i.e. cycles of pushing and pulling or twisting back and forth, for axial and shear test respectively), and the response is continuously measured. For a purely elastic material, the response to an applied force or strain is immediate. For a viscoelastic material, the response has a time delay and the magnitude (phase shift) of the delay is an indication of whether the material is more elastic (time delay is short) or viscous (time delay is long). For a purely viscous material, the response is 90° out of phase with the applied force or strain.

Alt-text: Text box 1

**Fig. 3:**
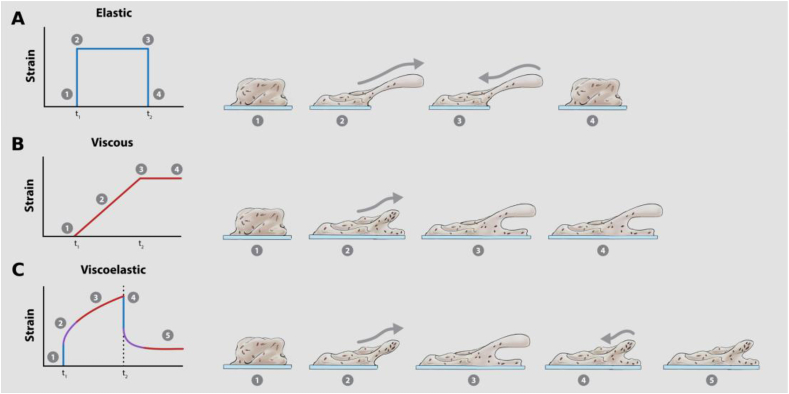
**Mechanical response of biofilms to a constant stress.** Creep-recovery curves are powerful analyses that can be performed using both spinning disc rheology and time-lapse microscopy of flow cell biofilms, that can determine biofilm mechanical behavior. During this analysis a constant stress is applied to the biofilm (t_1_) and the resulting deformation (strain) is measured. The stress is then removed (t_2_) and the recovery measured. The left panel depicts typical creep-recovery curves for materials with **(A)** elastic, **(B)** viscous and **(C)** viscoelastic properties. The right panels depict how biofilms with these properties would respond, at different stages of the analysis. **(A)** Biofilms that are predominately elastic (1), in response to an applied stress will instantly deform (2) and maintain this deformed state while the stress is applied (3). However, when the stress is removed the biofilm will instantly return to the original per-deformed state or structure (4). This response is akin to elastic bands being stretched. **(B)** Biofilms that are predominately viscous (1), in response to an applied stress display flow behavior (2), and will continuously deform and flow while the stress is applied (3). When the stress is removed, the biofilm will maintain the deformed state, and will not return to the original structure (4). This response is akin to honey being poured onto a surface. **(C)** Biofilms that are viscoelastic, display both elastic (blue), viscous (red) and a combination of both, where the elastic behavior begins to transition to viscous behavior (purple), which is typically time dependent. Viscoelastic biofilms (1), in response to an applied stress, will instantly deform due to an elastic response (2). Over time this behavior will transition to a viscous response and the biofilm will begin to deform by viscous flow (3). When the stress is removed the biofilm will show an initial elastic recoil (4), however, will never return to the original pre-deformed state due to the transition to viscous recovery (5). This response is akin to silly putty. (For interpretation of the references to colour in this figure legend, the reader is referred to the Web version of this article.)

During mechanical indentation analysis, in compression mode, the force required to compress a biofilm is measured. From the slope of the resulting force-displacement curve, the Young’s modulus (*E*) of the biofilm can be determined [11]. The Young’s modulus is a measure of the elasticity, or stiffness, of a material when exposed to a normal force [11]. Both spinning disc rheology and flow cell systems (Fig. 2B and C) can be used to measure changes in biofilm deformation, when exposed to constant or varying shear stresses. For spinning disc rheology, stresses that build up in the biofilm can also be measured when it is exposed to constant or varying strains or stresses. For both these mechanical methods, a common measurement is creep-recovery analysis, where the deformation of a material, in response to an applied stress, and the subsequent recovery when the stress is removed, is measured (Fig. 3). From this analysis the viscoelastic properties can be determined, as well as the shear modulus (*G*) and the viscosity (η). The shear modulus, analogous to the Young’s modulus determined from indentation analysis, describes the extent that a material can resist deformation when exposed to shear stress, and can be used to index the elastic behavior of a material [9].

Finally, spinning disc rheometry can also be used for *dynamic oscillation analyses*, where the stress or strain is cyclically oscillated at steady or changing frequency, in a sinusoidal manner (Fig. 2B). These analyses are commonly used for the analysis of viscoelastic materials, as they can describe complex behavior by determining the storage (G’) and loss (G”) moduli [9,10]. These moduli describe the stress response of a material when exposed to oscillatory shear. The storage modulus describes the elastic, solid-like behavior (energy that can be stored), and the loss modulus the liquid-like viscous behavior (energy that is lost) of a material, [10]. Dynamic oscillation analyses can also be used to determine the yield stress or strain, which is the point where the biofilm undergoes a structural change and begins to transition to a liquid [12]. Yield stress can be used as a surrogate indicator of strength, while the yield strain, an indicator for malleability.

Modeling can also be used to determine other viscoelastic parameters. Creep-recovery analysis is commonly modeled as a system of springs (the elastic elements) and dashpots (the viscous elements). One of the simplest systems is the two-element Burgers model, which consists of a spring and dashpot in series (a Maxwell element) and a spring and dashpot in parallel (a Kelvin–Voigt element). However, more elements can be incorporated to improve the model [13,14]. More advanced models have also been developed which are used to describe complex behavior of biofilms and how they deform and detach in response to shear [[15], [16], [17]].

An important consideration when mechanically testing complex viscoelastic materials, such as biofilms, is that the measured properties will be a function of the intrinsic material properties, and the testing conditions themselves. For example, moduli on the scale of Pa – kPa have been reported for biofilms [19]. This variation is likely due to differences in the organism, EPS content, growth conditions and mechanical methodology and parameters. Furthermore, the time scales over which measurements are performed will impact interpretations of mechanical properties. Under very short time scales (milliseconds - seconds) a material can behave as an elastic solid, whereas over longer time scales (minutes – hours) the time dependent viscous characteristics will become more prominent. It is therefore somewhat difficult to compare results across studies. Moving forward, it may be of interest for the field to develop standard protocols for mechanical analyses, to minimize this variation and permit direct comparisons to be made across studies. Despite this, moduli determined from rheological analyses are intrinsic properties that describes the resistance or compliance of a material when exposed to mechanical forces [9], and are powerful means to understand how biofilms interact with, and respond to mechanical changes in their surrounding environment.

There have been a number of reviews, which discuss and compare biofilm mechanics of different bacteria and analysis techniques. However, only a few have connected these concepts to applied settings, such as infection [20,21]. Here, we will review how broader biofilm mechanical properties may be of relevance to infection and biofilm survival.

## Biofilm viscoelasticity as a survival adaptation strategy

Due to the different methodologies that have emerged to analyze biofilm mechanics it is difficult to compare results between studies. In addition, mechanical properties are diverse across organisms, making it difficult to find a universal constitutive model. It is emerging that *viscoelasticity* is a trait common to biofilms, however they can also have other material properties. For example, biofilms have been described from viscoelastic, *poroelastic*, *elastoplastic* and *elastic solids* to *Newtonian liquids* and *Bingham fluids* (Text box 2; Fig. 3 [3,16,17,[22], [23], [24]]). Differentiating between, and decoupling these behaviors can be complicated and often requires computational modeling [16]. Mechanical analyses have been developed to separate poroelastic and viscoelastic properties of cross-linked polymer gels [25]. Adapting such analyses from the engineering field would further advance our understanding of biofilm mechanics and the complexity of this behavior. As viscoelasticity seems to be a trait common to biofilms, with prehistoric biofilm mats predicted to have been viscoelastic [26], it has been proposed that viscoelasticity emerged as a general survival adaptation for biofilms to withstand fluctuations in the surrounding environment, such as shear stresses [27].

Text box 2 *Viscoelastic:* Materials that display linear elastic behavior over short time scales and linear viscous behavior over long time scales.

*Poroelastic:* Elastic materials that have a pore network. The fluid-filled pores exert a pressure on the overall material, which influences the mechanical responses to external stresses and strains.

*Elastoplastic:* Materials that display linear elastic behavior in response to small stresses or strains, and plastic deformation in response to large stresses or strains. Plastic deformation is an irreversible change in shape. The reason for this is dependent on the type of material, i.e. metal, rock etc, however, for biological materials this is often due to cell rearrangements.

*Linear elastic*: Materials that display a linear relationship between stress and strain. The strain becomes zero immediately after stress release.

*Linear viscous (Newtonian)*: Materials that display a linear relationship between the stress and the strain rate i.e. the viscosity.

*Bingham fluid:* Viscous fluids that have a yield strength. Therefore, at stresses that do not exceed the yield, the material behaves as a solid. However, at stresses that exceed the yield stress the material displays viscous flow.

Alt-text: Text box 2

The viscoelasticity of biofilms appears to be an emergent behavior of the community. Single bacterial cells have a high Young’s modulus (kPa – MPa), indicating that they are mechanically stiff and rigid [19], whereas bacterial biofilms have a Young’s modulus on the order of Pa – kPa [19]. Furthermore, *Vibrio cholerae* biofilms composed solely of bacterial cells are mechanically weaker (reduced storage modulus, yield strain and yield stress) compared to wildtype biofilms with a full EPS [28]. Similarly, it was observed for *Pseudomonas fluorescens* biofilms that the cell-to-EPS ratio influences biofilm viscoelasticity, with more EPS conferring more ductile behavior [29], while *Streptococcus mutants* biofilms that have a higher cell density are stiffer and mechanically weaker compared to wildtype [30]. The interaction between EPS components also promotes biofilm cohesion, with stronger EPS interactions preventing biofilm mechanical failure [31]. These observations demonstrate that viscoelasticity is an emergent behavior of bacteria when they from biofilms, and that these properties are largely governed by EPSText box 2.

### EPS provides protection from mechanical stresses

EPS provides many essential functions to the biofilm [32]. One of these roles is protection from environmental stresses, such as desiccation and antimicrobials [32]. With the growing consensus that biofilm viscoelasticity is largely influenced by the EPS, we would argue that the EPS also serves to provide protection to withstand assault from mechanical shear.

It is now appreciated that individual EPS components offer unique mechanical properties to the biofilm. *Pseudomonas aeruginosa* can produce three different exopolysaccharides; alginate, Psl and Pel, which can make up the EPS, along with proteins and extracellular DNA (eDNA). Psl strengthens the biofilm and increases the elasticity, while Pel increases the ductility and malleability of the biofilm [33,34]. The elasticity imparted by Psl is dependent on the EPS protein CdrA, and predicted to be due, in part, to CdrA cross-linking the Psl polymers [34,35]. Wildtype *P. aeruginosa* produces little alginate. However, for mutants that overproduce this exopolysaccharide, the biofilms behave as viscoelastic fluids and have reduced viscosity and elasticity compared to wildtype [34,36,37].

Similarly, the EPS of *V. cholerae* biofilms is composed of Vibrio polysaccharide (VPS), and proteins RbmA, Bap1 and RbmC. Wildtype *V. cholerae* biofilms are more elastic (higher storage modulus) and stronger (higher yield stress) compared to biofilms of single (*vps*), double (*vps* and *rbmA*) and triple (*vps*, *rbmA* and *bap1*) EPS mutants. This was attributed to the EPS proteins cross-linking the VPS polymers [28]. Interestingly, Bap1 and RbmC show homology to RbmA and are predicted to perform partially redundant functions in the biofilm. Mechanically however, this is not the case as all three proteins were required for wildtype behavior [28].

These observations are also supported from modeling the stress response of bacterial biofilms, which identified multiple relaxation times that were attributed to different EPS components of the biofilm [14]. Together this suggests that it is mechanically advantageous for biofilms to produce multiple EPS components. This ensures that the biofilm is mechanically robust and can respond dynamically to external forces without detaching. Yan et al. [28] proposed that exposure to flow environments may have been a selective pressure driving the evolution of multiple EPS components, to ensure that the biofilm could withstand exposure to varying and increased shear stress.

## Biofilm mechanical responses to shear

### General mechanical responses to shear

**Fig. 4:**
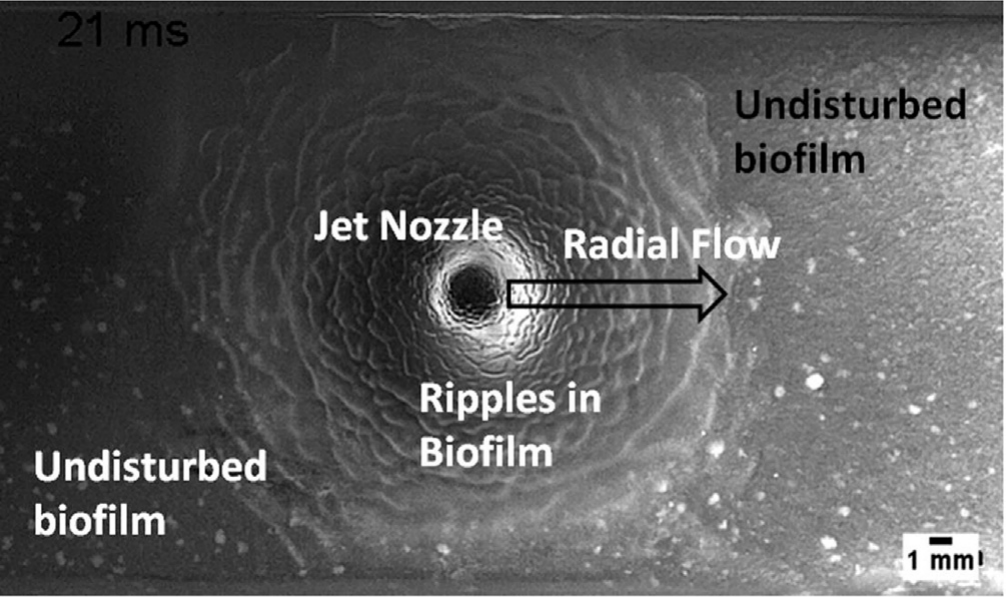
**High shear flows induce ripple formation in biofilms.** Here ripples formed in a *S. mutans* biofilm at 21 ​ms exposure to a high shear jet impingement of air ejected at a velocity of 42 ​m/s from a 1 ​mm diameter nozzle positioned perpendicularly 5 ​mm from the biofilm. Under these high shear conditions around the impingement site the biofilm was liquefied and flowed like water. Further detail of the experimental design can be found at [15,18].

Many biofilms are exposed to shear forces, particularly fluid flow, during their development, which impact biofilm structure and morphology. Biofilms grown under laminar flow (low shear) form heterogeneous cell clusters that are separated by water channels [38]. In contrast, biofilms that are grown under turbulent flow (high shear) form both cellular clusters, as well as ripple formations (Fig. 4). The cellular clusters are generally larger than those under laminar flow and are tapered in the direction of flow, forming streamers [38,39]. Interestingly, *P. aeruginosa* has been observed to undergo a form of mechanosensing that is sensitive to shear rate, termed rheosensing [40]. It was observed that a previously un-named operon, designated *fro* (flow-regulated operon), in response to high shear was induced 13-fold, compared to low shear [40]. It would therefore be interesting to explore how rheosensing influences biofilm development under inducing conditions, and to decouple this induction from other effects of high shear, such as increased metabolism (discussed below).

Biofilms also respond dynamically to fluctuations in fluid flow. When the shear stress is reduced the biofilm retracts, behaving as a viscoelastic solid. When exposed to higher stresses the biofilm extends and exhibits hysteresis, behaving as a viscoelastic fluid [3]. However, there is evidence to suggest that not all biofilms exhibit hysteresis when exposed to oscillating shear (over minutes), further pointing towards the diverse mechanical properties that biofilms display [41]. Modeling biofilm deformation, when exposed to increasing fluid flow, showed that upstream portions of the biofilm experience a compressive force, whereas the top of the biofilm experiences a tensile force [17]. Poroelastic simulations demonstrated that this coincided with a Young’s modulus gradient within the biofilm, due to changes in biofilm porosity. Increased flow resulted in reduced porosity and consolidation at the base of the biofilm. This portion of the biofilm displayed a higher Young’s modulus, and reduced deformation in response to shear. Higher regions of the biofilm had a lower Young’s modulus and showed greater deformation [17]. Furthermore, a numerical model of poroelasticity, coupled with mechanical analysis, showed that biofilm mechanical response could be described as elastic, for small irreversible deformations, or viscoelastic and elastoplastic, for permanent deformation [16]. Together this demonstrates that biofilms alter their mechanical properties in response to external forces, and that this response is highly dynamic. It has been proposed that an equilibrium exists between biofilm EPS and shear stresses in which the biofilm develops [3]. These observations have implications to infection and biofilm clearance, discussed below.

### Implications to removal

#### Increases in biofilm biomass

**Fig. 5:**
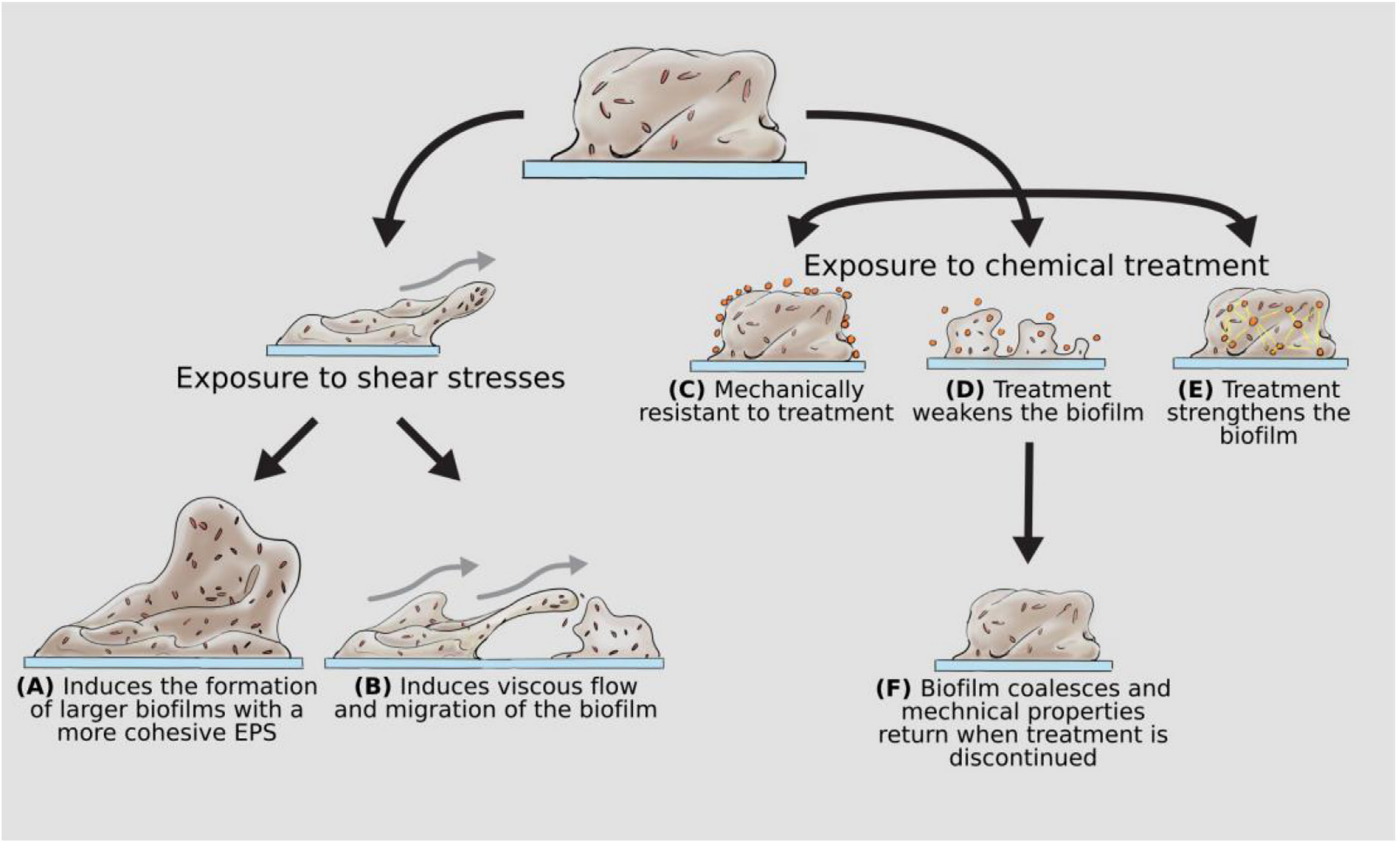
**Schematic of how biofilm mechanics can influence survival.** Summary of how biofilm viscoelasticity can protect the microbial community when exposed to **(A**–**B)** mechanical forces, such as shear stress, and **(C–F)** chemical treatment.

There is a general phenomenon that thicker biofilms, with increased biomass, develop when grown under high shear stresses (Fig. 5A [4,22,42,43]). This can generally be attributed to two complementary processes. Firstly, biofilms grown under high shear are more elastic and rigid, and resist mechanical failure and detachment to a greater extent, compared to biofilms exposed to low shear [4,22]. This suggests that in response to high shear the EPS becomes stronger. It has been proposed that high shear promotes the alignment of, and increased interactions between EPS components, leading to increased adhesion and cohesion [4].

Secondly, under high shear there is increased diffusion between the surrounding fluid and biofilm. When grown under flow conditions, due to the heterogeneous structure and EPS, fluid flow is slowed surrounding the biofilm, and largely arrested through the biofilm. The slow moving fluid prevents efficient diffusion into and out of the biofilm, and is referred to the mass transfer boundary layer [44,45]. Mass transfer boundary layers have been measured at 10 ​μm to over 1 ​mm, depending on the region of the biofilm and the organism [45]. As the fluid velocity increases, this boundary layer decreases [44,45]. Therefore, biofilms grown at higher shear experience increased diffusion of solutes and ultimately increased growth, compared to biofilms at low shear. In line with this, *Geobacter sulfurreducens* biofilms grown under high shear showed increased metabolic rates, compared to biofilms grown under low shear [46]. Interestingly, this increased metabolic rate came at a cost to biofilm viability over extended growth (7 days), presumably due to accumulation of waste byproducts in the biofilm [46].

These combined consequences of exposure to high shear stresses, over a period of hours - days, leads to the development of thicker biofilms that are highly adherent and mechanically robust (Fig. 5A). This would have important consequences to infections where biofilms experience high or fluctuating shear stresses, such as endocarditis or catheter associated urinary tract infections, by permitting survival of the community.

#### Viscous flow of the biofilm in response to high shear

In response to shear stresses (applied over minutes), wildtype and mucoid *P. aeruginosa* biofilms behave as viscoelastic fluids and exhibit viscous flow [4,37]. Viscous flow of biofilms across surfaces may therefore offer an unrecognized form of biofilm dissemination (Fig. 5B [4]). This could have important consequences in industry and infection, particularly implanted medical devices. Indeed, biofilms with flowing wave-like structures have been observed in the lumen of explanted endotracheal tubes. It was predicted that this patterning was evidence of viscous flow of the biofilm down the tube, resulting in ventilator-associated pneumonia [47].

Viscous flow could also offer a passive form of biofilm collective motion. In line with this, *Staphylococcus aureus* biofilms, under laminar flow, were observed to exhibit viscoelastic rolling along the length of a flow cell [27]. Microcolonies were observed to form EPS tethers to the underlying substratum, which over short time periods stretched elastically. Over longer time periods the tethers flowed and detached resulting in a forward rolling motion of the microcolony. A relaxation time of 12 ​min for this transition was determined [27]. The migration rate was slow, up to 55 ​μm/h [27], however, this passive form of migration would have important consequences for the colonization of surfaces, particularly for traditionally non-motile organisms. Along these lines, *Neisseria gonorrhoeae* colonies showed liquid-like behavior and ordering, mediated by the actions of type IV pili during twitching-mediated motility of the colony [48].

#### Resistance to mechanical clearance

Mechanical cleaning strategies are often used in industry and medicine to dislodge biofilms from surfaces. High-velocity sprays are one such method. Interestingly, exposure to high-velocity microsprays (applied over milliseconds - seconds) induced interfacial instabilities for *S. mutants, Staphylococcus epidermidis* and *P. aeruginosa* biofilms (Fig. 4 [23,49]). Due to the different EPS, the instabilities resulted in different physical interactions of the biofilms. Both *S. mutants* and *S. epidermidis* biofilms behaved as viscous liquids, forming migratory ripple-like structures (Fig. 4). These structures were transient and dissipated when the flow was stopped, after which the biofilm coalesced by viscoelastic recoil, within milliseconds [23,49]. In contrast, *P. aeruginosa* biofilms formed stationary wrinkle-like structures. The wrinkle structures are evidence of cohesive failure at the biofilm-substratum interface, rather than the transition to fluid behavior [23]. These observations suggest that the use of high-velocity sprays may serve to spread the biofilm across surfaces, rather than mechanically remove them. In support of this, biofilms which were disrupted by exposure to high shear, recovered their initial mechanical properties within minutes once the shear was removed [50].

Therefore, these methods of mechanical removal may not be as efficient at removing biofilm as originally appreciated. This would have significant implications in medicine, particularly dental hygiene where high-velocity sprays are often used. Interestingly, polypropylene surfaces coated with a pliable polyacrylamide hydrogel exhibit reduced colonization by *Escherichia coli*, compared to uncoated surfaces [51]. Vibrating the surfaces (minutes) at the structural resonance frequency of the hydrogel coating resulted in increased *E. coli* biofilm removal, whereas the uncoated surface showed little removal. This vibration also removed β-linked polysaccharides [51]. This suggests that specific high oscillations of synergic materials can mechanically remove adhered bacteria. The development of such materials would have significant implications in the development of medical devices, which are often infected by biofilms.

Host innate immune systems also employs mechanical clearance mechanisms to remove microorganisms and biofilms to prevent their establishing infections. One such example is the mechanical clearance of mucus from the lung, and subsequently inhaled microorganisms and particles, by mucociliary and cough clearance. The mucus viscoelasticity ensures efficient clearance by both mechanisms. In disease states, such as cystic fibrosis (CF), mucus overproduction and changes in mucus composition and viscoelasticity, results in inefficient clearance and accumulation of mucus in the lung [52]. It has been hypothesized that the EPS of infecting biofilms in the mucus layer can contribute locally to mucus viscoelasticity, further impeding mucus clearance, promoting biofilm persistence [37]. In support of this hypothesis, eDNA is a main component of CF sputum, forming thick bundles that do not associate with mucin polymers [53]. This eDNA is largely of human origin [54], and is presumably released during inflammatory cell death or NETosis. NETosis (neutrophil extracellular traps) is the process whereby neutrophils extrude their cellular DNA content, coated with antimicrobial enzymes and proteins, that trap and kills microorganisms [55]. The presence of eDNA significantly increases CF sputum viscoelasticity, and subsequently increased eDNA concentrations is associated with poorer lung function, presumably because the mucus is more difficult to clear [[52], [53], [54],^56^]. Similarly, CF sputum contains substantial levels of biofilm EPS polysaccharides. For example alginate, the dominant exopolysaccharide produced by *P. aeruginosa* mucoid isolates, is present in CF sputum at concentrations of 1–100 ​μg/mL [57]. This is equivalent to levels quantified from mucoid monocultures *in vitro* [58]. There is also evidence to suggest that CF sputum infected with different bacteria have different viscoelastic properties [59].

Along these lines, it has been postulated that changes in CF mucus viscoelasticity, due to extensive amounts of eDNA and biofilm EPS, retards neutrophil migration through the mucus layer to the infecting biofilms [20]. In support of this, it has been observed that neutrophils displayed little migration in thickened mucus representative of that produced during CF [60]. Similarly, it has been proposed that biofilm mechanics may facilitate resistance to phagocytic clearance by neutrophils [20,34]. It was suggested that a combination of biofilm size and cohesive strength determines the extent that a biofilm will be phagocytized, and that above a threshold biofilms may resist mechanical attack by immune cells [20].

Other methods of mechanical clearance of biofilms include wound debridement. Debridement is a standard care for wound management, and involves the physical removal of infected and dead tissue to promote healing. Debridement techniques include mechanical debridement using surgical removal, gauze, lavage or ultrasound, and autolytic or chemical debridement. After debridement, antimicrobial dressings are applied to help reduce biofilm and move the wound onto healing [61]. Despite these methods being temporarily successful at reducing microbial burden in the wound, the biofilm re-establishes within 24–48 ​h [62,63], suggesting that biofilm remained despite frequent and aggressive forms of physical removal attempts. It would be of interest to explore how biofilm viscoelasticity contributes to this resistance to removal when exposed to such mechanical clearance strategies.

### Exploiting biofilm properties to enhance mechanical removal

By understanding biofilm viscoelasticity, these properties can be exploited to design more efficient strategies for their mechanical removal. Wildtype *V. cholerae* biofilms are non-polar and hydrophobic. It was demonstrated that immersing the biofilm in water could peel the biofilm off hydrophilic surfaces, by inducing interfacial fractures at the biofilm-substrate interface [28]. An immersion rate of 0.1 ​mm/s was observed to be optimal at removing the biofilm, with faster rates reducing the peeling efficiency, as water was able to pass over the biofilm. This has been observed for agar, paper, membrane and metal surfaces, as well as for *P. aeruginosa* biofilms [28]. This has vast implications in industry and medical applications, and suggests that a slow immersion may be a more successful method of mechanical removal, rather than high-velocity sprays, as is the general practice.

Furthermore, material properties of the underlying substratum have been shown to influence the biofilm’s mechanical properties. *S. mutants* biofilms grown on hydrophilic surfaces (hydroxyapatite and titanium) were less stiff (lower Young’s modulus) compared to biofilms grown on hydrophobic surfaces (stainless steel and polyethylene) [64]. This suggests that medical devices could be designed, which promote the formation of biofilms with known mechanical properties. By exerting some control over the biofilm’s mechanical properties would facilitate the development of strategies to aid their removal once they develop.

## Biofilm mechanical response to chemical and antimicrobial exposure

As biofilms are mechanically diverse, so too are their mechanical response to exposure of different molecules and chemicals (Fig. 5). For example, nutrient availability influences biofilm mechanics [[65], [66], [67]]. Growth media influences the amount, and composition, of EPS produced during biofilm formation. *P. fluorescens* biofilms grown under low nutrient conditions were stiffer (higher Young’s modulus) and more adhesive (higher adhesion force and work of adhesion) compared to biofilms grown in high nutrient conditions. It was predicted that biofilms produce less EPS under lower nutrient conditions, accounting for the different biofilm mechanics [65]. Similarly, *P. aeruginosa* mucoid biofilms grown in artificial sputum media (ASM) have increased eDNA, compared to biofilms grown in LB. Furthermore, mucoid biofilms grown in ASM, supplemented with mucin, have increased exopolysaccharide, compared to biofilms grown in ASM, without mucin, or LB [67]. These changes in EPS content subsequently led to different architectures and stress-relaxation profiles of biofilms grown in the different media [67]. Mucoid biofilms grown in ASM, supplemented with mucin, had a heterogeneous architecture, and slow stress-relaxation profile, due to the increased abundance of exopolysaccharides, both soluble and insoluble, and eDNA [67]. Finally, different growth media influences how bacterial cells interact with artificial hydrogels, where the presence of tryptone allowed *S. epidermidis* and *E. coli* cells to cross-link directly with agarose polymers [66].

Exposure to different metal ions has a drastic impact on biofilm mechanics, presumably through electrostatic interactions with the biofilm EPS. Exposure to monovalent cations (Na^+^, K^+^) typically does not influence biofilm mechanics [50,68,69]. However, for *S. epidermidis* biofilms, treatment with low and high concentrations of NaCl weakened the biofilm (reduced both shear modulus and viscosity) [69,70]. In contrast, treatment with intermediate NaCl concentrations stiffened the biofilm (increased storage and loss modulus) [70]. It was suggested that NaCl initially stimulates the production of EPS components, accounting for the increased stiffness from low to intermediate NaCl concentrations. However, at higher concentrations the ions may decouple the interactions between the cells and EPS, weakening the biofilm [70]. Therefore, the mechanical response to monovalent metal ions is varied between bacterial species and the ion concentrations, presumably through different interactions between the metal ions and biofilm EPS, however this warrants further investigation, since metal salts can influence a number of factors including cross-linking, osmotic stress and bacterial activity.

Exposure of biofilms to divalent metal ions induces an array of mechanical responses, which appear to be specific to the metal ion, as cations of the same valency can induce different responses. For example, wildtype *P. aeruginosa* biofilms exposed to Fe^2+^ become less elastic (decreased storage modulus). However, treatment with Ca^2+^ or Cu^2+^ does not influence the mechanics, suggesting that electrostatic interactions alone are not responsible for influencing the mechanical properties [50]. Psl and Pel, the dominant polymers of nonmucoid *P. aeruginosa* EPS, are neutral and positively charged respectively [71,72], and therefore unlikely to interact electrostatically with divalent cations. Rather it appears that Psl is cross-linked by the EPS protein CdrA [34,35], and that Pel is cross-linked by eDNA [71]. However, there is evidence that Psl can bind and sequester Fe^2+^ (and Fe^3+^) [73], which may account for the specific effect of Fe^2+^, although this appears to be to the detriment of biofilm mechanics. Similarly, *Bacillus subtilis* biofilms treated with Cu^2+^, Zn^2+^ and to a lesser extent Fe^2+^, resulted in increased elasticity of the biofilm (increased storage modulus), whereas treatment with Mg^2+^ or Ca^2+^ had no influence [68]. Again, this suggests that ionic cross-linking alone does not account for these effects, but that there are specific interactions between EPS components and metal ions. In contrast, treatment of *P. aeruginosa* mucoid biofilms with divalent cations (Ca^2+^, Mg^2+^ and Fe^2+^) resulted in stiffer biofilms (increased shear modulus) [69]. Alginate is a negatively charged polymer, and therefore appears to be readily cross-linked by multivalent cations [36,50,69,74]. However, certain divalent cations (Ca^2+^, Mn^2+^ and Mg^2+^) can induce the production of alginate in *P. aeruginosa* [74,75], and therefore increased amounts of alginate could also be influencing the mechanics in the presence of these ions.

Treatment of *P. aeruginosa* biofilms (wildtype and mucoid) and *B. subtilis* biofilms with trivalent ions (Fe^3+^ and Al^3+^) resulted in increased stiffness of the biofilm (increased elasticity, viscosity and storage modulus) [50,68,69]. However, treatment of *S. epidermidis* biofilms with Fe^3+^ had no influence on biofilm mechanics [69]. Therefore, exposure to trivalent cations, where it has an effect, appears to consistently lead to increased stiffness of the biofilm. This is compared to divalent cations, where both biofilm weakening and strengthening effects have been observed.

Therefore, different types and concentrations of metal ions can have varied impacts on biofilm mechanics (Fig. 5). This appears to be dictated by different physical interactions, as the observed changes were on the order of seconds, suggesting that they are generally not metabolic responses [50,69]. These observations could have important consequences for biofilm survival in both the natural environment and in an infection, where biofilms are can be exposed to high salt concentrations [76]. In support of this, the stiffening effect of Cu^2+^, Fe^3+^ and Al^3+^ on *B. subtilis* biofilms appeared to be protective against shear induced erosion [68].

### Mechanism of tolerance to biocides and antimicrobials

Interestingly, exposure to many common antimicrobials that are used to kill or disrupt biofilm bacteria do not alter the mechanical properties of the biofilm, suggesting that these communities are mechanically resistant and robust in the face of these agents. *P. aeruginosa* wildtype biofilms are mechanically resistant to a diverse range of chemicals (gentamicin, colistin, ofloxcin, salicylic acid, amylase, ethanol, triton, bleach, urea, xylitol and hexanediol) [50,69]. However, treatment with citric acid, chelating agents, or in the case of mucoid biofilms glutaraldehyde and ciprofloxacin, weakened the biofilm (reduced storage modulus and viscosity, and increased peak strain) [50,69]. Conversely, treatment of *S. epidermidis* biofilms with rifampin, urea and chlorine weakened the biofilm (reduced elasticity and viscosity, increased peak strain), while treatment with glutaraldehyde, nisin and ammonium had no influence on the biofilm mechanics [69,77]. The weakening of chlorine treated *S. epidermidis* biofilms appeared to be a concentration-dependent effect, as higher concentrations led to increased biofilm removal [77]. Similarly, treatment of *E. coli* biofilms with increasing concentrations of chlorhexidine, resulted in increasing biofilm elasticity (increased spring constant). Biofilm elasticity also increased with longer exposure times (0–40 ​min). The increased elasticity was attributed to precipitation of intracellular material, induced by chlorhexidine [78]. However, concentration-effects of other microbial agents mentioned above have not been described and warrants further investigation in the field.

This highlights the difference between biofilm removal and killing, as a number of these agents were able to reduce viability of the encased cells, however had no effect on biofilm mechanics [77]. Furthermore, *P. aeruginosa* biofilms are able to recover their mechanical properties after exposure to chemicals that weakened the biofilm (citric acid and EDTA) [50]. This suggests that when chemical eradication strategies are discontinued, remaining biofilm can coalesce and surviving bacteria can re-populate the structure, facilitating the recalcitrance of these communities (Fig. 5 [50]). Finally, mature (84 ​h) *P. aeruginosa* biofilms are resistant to degradation by DNaseI [79]. This suggests that for mature biofilms, the EPS may be protected from chemical degradation due to stronger interactions and cross-linking between neighboring EPS components. Similarly, biofilms with a more complex EPS, specifically increased amounts of insoluble exopolysaccharides and eDNA, display increased tolerance to antimicrobials [67]. These observations may influence the design and efficacy of new therapies that are being developed, which aim to target the biofilm EPS to weaken or degrade the community.

### Exploiting mechanical properties to chemically eradicate biofilms

Alternative approaches to therapies that weaken and degrade the biofilm are those that exploit biofilm mechanics to facilitate increased antimicrobial access into the biofilm. Biofilms under a normal force display a J-shaped stress-strain relationship [3,37,64]. At low stresses, materials with a J-shaped curve easily stretch due to the tortuosity of loose polymer entanglements. Deformation at these stresses is reversible and the energy used to stretch the material is released once the load is removed. Modeling indicates that water in a biofilm is the fastest relaxation component (the fastest Maxwell element), and will flow out of the biofilm under low stresses [14]. This suggests that under non-destructive normal forces, water can easily be compressed from the biofilm, and drawn back into the structure, once the force is removed. In support of this, *S. mutans* biofilms exposed to osmotic stress (treatment with PEG-8000) became stiffer (increased Young’s modulus) [64]. It was hypothesized that PEG-8000 treatment drew water from the biofilm, which altered the osmotic equilibrium and altered the mechanical properties [64]. Similarly, compression of biofilms grown in membrane fouling simulators was associated with increased hydraulic resistance. This was due to structural deformation and reduced porosity of biofilms under compression, and subsequently reduced permeability. The structural deformation and porosity were recovered under relaxation [16]. This behavior has the potential to be exploited to aid the penetration of antimicrobials into the biofilm. By compressing the biofilm, antimicrobials could be drawn into the biofilm upon removal of the compressive force. This would be an interesting avenue of research and could have many applications in medicine, particularly in wound healing, where negative pressure wound dressings, which apply a pressure to the wound, are routinely used.

## Conclusions

The field of biofilm mechanics has advanced extensively in the last decade (Fig. 1). However, there is still a limited understanding of how biofilm mechanics and viscoelastic principles relate to, and facilitate biofilm survival. It is evident that while biofilms are viscoelastic, they are mechanically diverse. However, it appears that an elastic relaxation time of approximately 18 ​min is a mechanical trait common to biofilms [80]. The elastic relaxation time is the time taken for a material to release absorbed stresses and for the material to relax. It would be interesting to explore if this conserved trait could be exploited to develop novel therapies for biofilm removal. Other interesting areas still to explore include the mechanical properties of purified EPS components, and how the mechanics differ between mixed community and mono-species biofilms. This will lead to a better understanding of how these properties change with the collective behavior of the biofilm. Comparing the viscoelasticity of infected versus inflamed tissue and fluids would also be an interesting area to explore, and could lead to rheology being used as a diagnostic tool to differentiate between these two states.

Finally, it appears that common to different modes of biofilm growth (air-surface and air-liquid interfaces) internal mechanical stresses develop due to cellular growth. Buckling of the biofilm dissipates these stresses, relaxing the biofilm and resulting in the formation of extensive wrinkle structures [37,[81], [82], [83], [84]]. Studying and modeling these patterns has been the focus of intense investigation in the field. While these are interesting from a physics and basic biology standpoint, from an applied perspective, a shift to studying the internal stresses may be warranted. It has been proposed that the internal stresses may be a self-healing mechanism for the biofilm [83]. When pellicle biofilms were mechanically damaged, releasing the internal stresses, localized expansion of the biofilm occurred resulting in recovery of the damaged region. It was observed that a *B. subtilis* pellicle could expand up to 20% of its size after ablation [83]. Therefore, studying these observations in more relevant models would be interesting to determine if this facilitates biofilm healing and expansion in other settings.

Here we have attempted to implicate biofilm mechanics specifically to survival in infection. We postulate that viscoelasticity promotes biofilm survival and expansion under shear, and resists mechanical and chemical clearance, in line with previous reviews (Fig. 5 [20,21]). We therefore propose that viscoelasticity contributes to the virulence of biofilms during infection, promoting the persistence and recalcitrance of these communities.

## Declaration of competing interest

Authors declare no competing interests.
